# Persistence of attenuated HIV-1 *rev *alleles in an epidemiologically linked cohort of long-term survivors infected with *nef*-deleted virus

**DOI:** 10.1186/1742-4690-4-43

**Published:** 2007-07-01

**Authors:** Melissa J Churchill, Lisa Chiavaroli, Steven L Wesselingh, Paul R Gorry

**Affiliations:** 1 The Macfarlane Burnet Institute for Medical Research and Public Health, Melbourne, Victoria, Australia; 2 Department of Microbiology and Immunology, University of Melbourne, Melbourne, Victoria, Australia; 3 Department of Medicine, Monash University, Melbourne, Victoria, Australia

## Abstract

**Background:**

The Sydney blood bank cohort (SBBC) of long-term survivors consists of multiple individuals infected with *nef*-deleted, attenuated strains of human immunodeficiency virus type 1 (HIV-1). Although the cohort members have experienced differing clinical courses and now comprise slow progressors (SP) as well as long-term nonprogressors (LTNP), longitudinal analysis of *nef*/long-terminal repeat (LTR) sequences demonstrated convergent *nef*/LTR sequence evolution in SBBC SP and LTNP. Thus, the *in vivo *pathogenicity of attenuated HIV-1 strains harboured by SBBC members is dictated by factors other than *nef*/LTR. Therefore, to determine whether defects in other viral genes contribute to attenuation of these HIV-1 strains, we characterized dominant HIV-1 *rev *alleles that persisted in 4 SBBC subjects; C18, C64, C98 and D36.

**Results:**

The ability of Rev derived from D36 and C64 to bind the Rev responsive element (RRE) in RNA binding assays was reduced by approximately 90% compared to Rev derived from HIV-1_NL4-3_, C18 or C98. D36 Rev also had a 50–60% reduction in ability to express Rev-dependent reporter constructs in mammalian cells. In contrast, C64 Rev had only marginally decreased Rev function despite attenuated RRE binding. In D36 and C64, attenuated RRE binding was associated with rare amino acid changes at 3 highly conserved residues; Gln to Pro at position 74 immediately N-terminal to the Rev activation domain, and Val to Leu and Ser to Pro at positions 104 and 106 at the Rev C-terminus, respectively. In D36, reduced Rev function was mapped to an unusual 13 amino acid extension at the Rev C-terminus.

**Conclusion:**

These findings provide new genetic and mechanistic insights important for Rev function, and suggest that Rev function, not Rev/RRE binding may be rate limiting for HIV-1 replication. In addition, attenuated *rev *alleles may contribute to viral attenuation and long-term survival of HIV-1 infection in a subset of SBBC members.

## Background

The Sydney blood bank cohort (SBBC) of long-term survivors (LTS) consists of multiple individuals who became infected with attenuated strains of human immunodeficiency type 1 (HIV-1) via contaminated blood products from a common blood donor between 1981 and 1984 [1-3]. Long-term prospective studies showed convergent evolution of *nef*/long-terminal repeat (LTR) sequences in virus harbored by SBBC members, characterized by progressive sequence deletions toward a minimal *nef*/LTR structure retaining only sequence elements required for viral replication [4]. Thus, gross deletions in the *nef*/LTR region of the HIV-1 genome contribute to viral attenuation and slow progression of HIV-1 infection in SBBC members. Despite convergent *nef*/LTR sequence evolution, after 22 to 26 years of infection SBBC members comprise antiretroviral therapy (ART)-naïve long-term nonprogressors (LTNP) as well as slow progressors (SP) who eventually commenced ART, suggesting that other viral and/or host factors may contribute to the *in vivo *pathogenicity (or lack thereof) of SBBC HIV-1 strains [3,4].

Numerous viral and host factors have been shown to affect the rate of HIV-1 disease progression [reviewed in [5-7]]. Viral genetic factors other than *nef*/LTR associated with SP or LTNP include mutations in the HIV-1 *gag*, *rev*, *vif*, *vpr*, *vpu *and *env *genes [8-13]. Host genetic factors linked to a delay in the onset of AIDS and prolonged survival include the CCR5 Δ32 mutation, CCR2-V64I polymorphism, and certain HLA haplotypes [14-17].

HIV-1 Rev is a 116 amino acid (aa), ~18 kD regulatory protein whose primary function is to mediate the nucleocytoplasmic transport, and therefore expression, of unspliced and singly spliced HIV-1 mRNA transcripts encoding viral structural proteins, via binding to the Rev response element (RRE) which is a complex RNA stem-loop structure present in these transcripts [reviewed in [[18-21]]. Therefore, Rev activity is essential for HIV-1 replication. Extensive mutational analysis of Rev has identified 2 distinct functional domains [reviewed in [21]]. These include an arginine-rich N-terminal region at aa positions 34 to 50 which contains the nuclear localization signal (NLS) and the RNA-binding domain (RBD) that mediates direct binding of Rev to the RRE, and a highly conserved leucine-rich C-terminal activation domain at aa positions 75 to 83 which contains the nuclear export signal (NES). The N-terminal NLS/RBD is flanked on both sides by less well defined sequences that are required for multimerization [22-25].

A previous study of *rev *alleles isolated from a subject with long-term nonprogressive HIV-1 infection showed a persistent Leu to Ile change at position 78 in the activation domain which attenuated Rev function and HIV-1 replication capacity [10], providing the first evidence that defective *rev *alleles may contribute to long-term survival of HIV-1 infection in some patients. A subsequent study of naturally occurring *rev *alleles with rare sequence variations in the activation domain showed variable reductions in Rev activity [26], although it was unclear from this study whether the reductions in Rev activity observed would be sufficient to attenuate HIV-1 replication capacity. In the present study, we undertook a genetic and functional analysis of HIV-1 *rev *alleles isolated from 4 SBBC subjects to determine whether defects in viral genes other than *nef*/LTR contribute to attenuation of HIV-1 strains harbored by SBBC members.

## Results and Discussion

### Subjects

The clinical history of the study subjects, results of laboratory studies and antiretroviral therapies have been described in detail previously [3,4,27]. The results of laboratory studies relevant for the longitudinal samples used in this study are summarized in Table 1. Briefly, D36 acquired HIV-1 sexually in December 1980. C18, C64 and C98 acquired HIV-1 by receiving blood products donated by D36 in August 1983, April 1983 and February 1982, respectively. After 19 years of asymptomatic infection without ART, D36 was placed on highly active ART (HAART) in January 1999 after evidence of HIV-1 progression. C98 was also placed on HAART in November 1999 after 18 years of HIV-1 infection, and died of causes unrelated to HIV-1 in March 2001. C64 has been infected for 24 years without ART, and has stable CD4 T-cells and below detectable viral load. C18 died of causes unrelated to HIV-1 in November 1995, but prior to death was asymptomatic with stable CD4 T-cell count for 12 years without ART. Thus, D36 and C98 are SP, and C18 and C64 are LTNP [3,4]. CCR5Δ32 genotyping by PCR showed that all subjects carried CCR5 (wt/wt) alleles ([28], and J. S. Sullivan, personal communication). CCR2-64I genotyping by PCR-RFLP showed that C64 and C98 carried the CCR2-64I (wt/wt) genotype [28]. The CCR2-64I genotype of C18 and D36 has not been determined.

**Table 1:** Subjects, longitudinal blood samples and corresponding laboratory studies.

**Subject**	**Date infected**	**Date of blood sample**	**CD4+ T-cells**^a ^**(cells/μl)**	**Viral load **^b ^**(RNA copies/ml)**	**HIV-1 progression status**^c^	**No. Rev clones sequenced**^d^
D36	12/1980.	5/1995	N/A	1400	SP	10
		1/1997	367	3200		10
		7/1999	N/A	BD		10
		4/2001	476	BD		10

C18	8/1983	12/1993	809	N/A	LTNP	10

C64	4/1983	8/1996	925	BD	LTNP	10
		8/1997	805	BD		10
		4/1999	1026	BD		10
		5/2000	875	BD		10

C98	2/1982	10/1995	576	670	SP	10
		2/1997	629	770		10
		11/1999	646	690		10
		5/2001	527	760		10

a; CD4+ T-cells were measured by flow cytometry.

b; Plasma HIV-1 RNA was measured by COBAS Amplicor HIV-1 Monitor Version 1.0 (Roche Molecular Diagnostic Systems, Branchburg, N.J.) prior to July 1999 and Version 1.5 after July 1999. HIV-1 RNA levels < 400 copies/ml (Version 1) or < 50 copies/ml (Version 1.5) were considered below detection.

c; The clinical status of the subjects has been described in detail previously [3, 4, 27].

d; The consensus sequences of the 10 Rev clones sequenced from each time point are shown in Additional file 1.

BD, below detection; N/A, not available; SP, slow progressor; LTNP, long-term nonprogressor.

### Persistence of unique *rev *alleles in SBBC members

**Figure 1:**
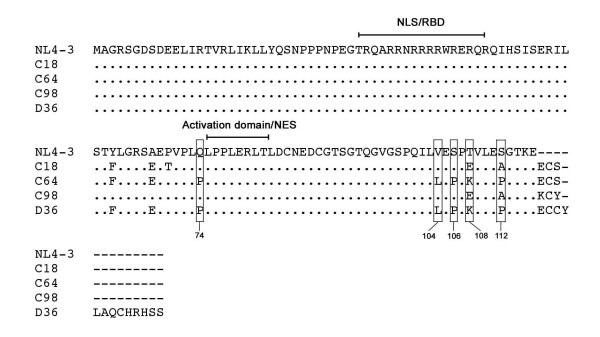
**Amino acid sequences of persistent and dominant SBBC *rev *alleles**. The HIV-1 Rev amino acid sequences shown represent those derived from the dominant and persistent *rev *alleles harboured by SBBC subjects C18, C64, C98 and D36. They are the consensus sequences of multiple independent Rev clones that persisted over a 4- to 6-year period in C64, C98 and D36, or which were dominant in a single blood sample obtained from C18 [see Additional file 1]. Amino acid alignments are compared to Rev from HIV-1_NL4-3_. Dots indicate residues identical to HIV-1_NL4-3 _Rev, and dashes indicate gaps. Boxed residues indicate amino acid substitutions which discriminate C18 and C98 Revs from C64 and D36 Revs. NLS; nuclear localization signal, RBD; RNA binding domain, NES; nuclear export signal.

Peripheral blood mononuclear cells (PBMC) isolated from blood samples longitudinally collected on 4 occasions between 1995 and 2001 were available from D36, C64 and C98 for this study (Table 1). Only one blood sample collected in 1993 was available from C18. Blood was taken from subjects in accordance with guidelines endorsed by the Australian Red Cross Blood Service human ethics committee. Multiple, independent full-length Rev clones containing the first and second Rev coding exons were generated from genomic DNA of each PBMC sample and sequenced. Phylogenetic analysis showed that all Rev sequences were clade B (data not shown). The dominant Rev aa sequence from each PBMC sample, which represents the consensus sequence from 10 independent clones, is shown in Additional file 1. In each subject where longitudinal PBMC samples were available (D36, C64 and C98), the persistence of a dominant *rev *allele was evident over a 4- to 6-year period. Figure 1 shows an aa sequence alignment of these dominant and persistent *rev *alleles as well as the dominant *rev *allele in the single C18 PBMC sample. Single aa changes at positions 74, 104, 106, 108 and 112 in sequence encoding Rev exon 2 segregated the dominant C18 and C98 Revs from the dominant C64 and D36 Revs. However, each dominant Rev sequence contained unique, distinguishing aa changes. In addition, C18, C64 and C98 Revs had a 3 aa extension at the Rev C-terminus, and D36 Revs had a 13 aa extension at this position. Similar C-terminal extensions were not identified in 164 Rev sequences available in the Los Alamos data base and other published studies [10]. Thus, the dominant and persistent Revs harbored by these SBBC members are unique. The following studies functionally characterized Rev proteins derived from these dominant and persistent SBBC *rev *alleles.

### Rev proteins derived from subjects C64 and D36 have attenuated RRE binding capacity

**Figure 2:**
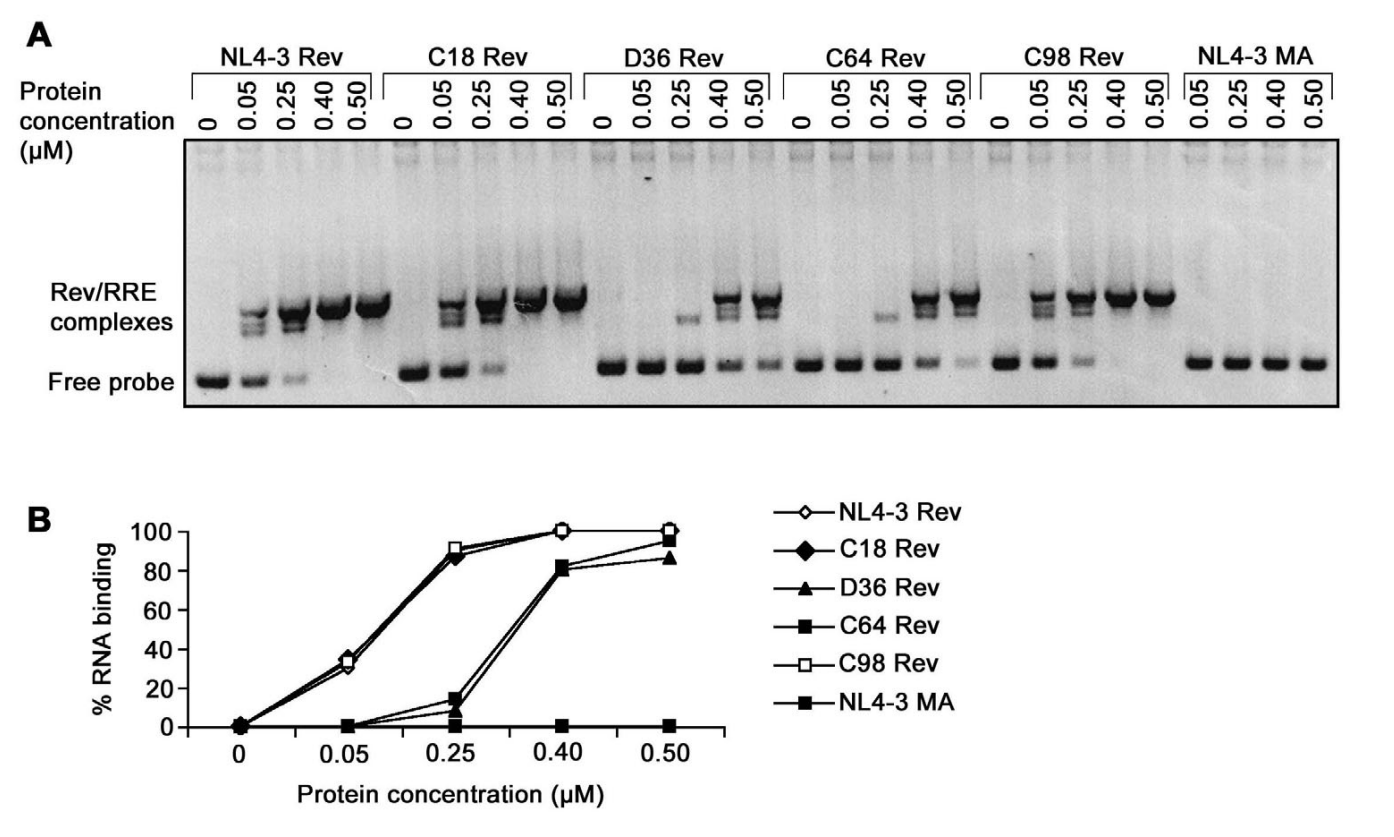
**Analysis of Rev/RRE binding**. RNA binding assays were conducted with [^32^P]-labelled RRE riboprobes and increasing concentrations of His-tagged Rev proteins, as described in Materials and Methods. Binding reactions containing increasing concentrations of His-tagged Matrix protein from HIV-1_NL4-3 _were included as negative controls. Rev/RRE complexes were resolved by electrophoresis in 5% (wt/vol) native polyacrylamide gels and visualized by autoradiography (A). Bands were quantified by phosphorimager analysis, and the percentage of RNA binding was calculated by dividing the signal intensity of bands associated with Rev/RRE complexes by the signal intensity of all bands, and multiplying this number by 100 (B). The data shown are representative of three independent experiments. *p < 0.01, Student's *t *test.

The ability of His-tagged Rev proteins derived from the dominant and persistent SBBC *rev *alleles to bind the RRE was quantified by electrophoretic mobility shift assays with [^32^P]-labelled RNA transcripts bearing the RRE (Fig. 2A). His-tagged Rev and Matrix proteins derived from HIV-1_NL4-3 _were used as positive and negative controls, respectively. Compared to His-tagged Rev from HIV-1_NL4-3_, the ability of His-tagged Revs from D36 and C64 to form Rev/RRE complexes at non-saturating Rev concentrations (0.25 μM) was reduced by approximately 90% (Fig. 2B). In contrast, the ability of His-tagged Revs from C18 and C98 to form Rev/RRE complexes at non-saturating Rev concentrations was similar to His-tagged Rev from HIV-1_NL4-3_. These results indicate that Rev proteins derived from the dominant and persistent D36 and C64 *rev *alleles have attenuated ability to bind the RRE.

### Rev amino acid sequences associated with attenuated RRE binding

Attenuated RRE binding was not due to mutations in the N-terminal RBD, since the amino acid sequences across this region were conserved among all SBBC *rev *alleles and were identical to HIV-1_NL4-3 _(Fig. 1). This was somewhat surprising, since previous studies showed that the RBD of Rev was the principal determinant of RRE binding [23-25,29-32]. The C-terminal 3 aa extensions present in C18, C64 and C98 Revs (Fig. 1) had no effect on RRE binding, since RRE binding by C18 and C98 Revs was similar to HIV-1_NL4-3_. Three amino acid changes that were conserved among D36 and C64 *rev *alleles and that were not present in C18 and C98 *rev *alleles were identified outside the RBD; Gln to Pro at position 74 immediately N-terminal to the Rev activation domain, and Val to Leu and Ser to Pro at positions 104 and 106 at the Rev C-terminus, respectively (Fig. 1). Amino acid changes also occurred at positions 108 and 112 which segregated C64 and D36 Revs from C18 and C98 Revs, but database analysis showed that amino acid variation is frequent at these positions (data not shown). Thus, amino acid changes at positions 108 and 112 are not likely to affect Rev/RRE binding. In contrast, the clade B consensus residues Gln-74, Val-104 and Ser-106 are normally highly conserved, with residue frequencies of 0.90, 0.94 and 0.97, respectively (Table 2). Pro-74, Leu-104 and Pro-106 are rare amino acid changes among clade B Revs; Only 16 *rev *alleles from 164 sequences available in the Los Alamos data base and other published studies [10] had Pro-74, Leu-104 or Pro-106, with individual residue frequencies of 0.049, 0.018 and 0.018, respectively (Table 2). The frequency of any 2 of these residues being present was 0.006. None of the available sequences had all 3 amino acid changes. Thus, the amino acid changes occurring in D36 and C64 Revs are unique. However, the presence of one or more of these amino acid changes was not able to discriminate between subjects with progressive or non-progressive HIV-1 infection (Table 2). Moreover, none of these amino acid changes occurred in a previously identified LTNP with defective *rev *alleles [patient MA [10], Table 2]. Thus, the contribution of any or all of these mutations to decreased RRE binding by D36 and C64 Revs, and possibly to slow or absent HIV-1 progression, is likely to be context dependent. Further mutagenesis studies are required to determine the contribution of Pro-74, Leu-104 or Pro-106 to diminished RRE binding by these Rev variants.

**Table 2:** *rev *alleles with rare Pro-74, Leu-104 and/or Pro-106 mutations^a^

**Clade B HIV-1 strain or Rev clone**	**Residue at position: (Clade B consensus residue; frequency**^b^**)**			**Frequency of residue combination**^b^	**Status of HIV-1 progression**^d^	**GenBank accession no**.	**Reference**
					
	**74 (Gln; 0.90)**	**104 (Val; 0.94)**	**106 (Ser; 0.97)**				
C64	**Pro**	**Leu**	**Pro**	unique	LTNP	EF634155	This report
D36	**Pro**	**Leu**	**Pro**	unique	LTS	EF634154	This report
MA^c^	Gln	Val	Ser	0.884	LTS	N/A	Iversen et al., [10]
C42	**Pro**	Val	Ser	0.049	N/A	AF538305	Unpublished
D31	**Pro**	Val	Ser	0.049	N/A	U43096	Kreutz et al., [48]
UKR1216	**Pro**	Val	Ser	0.049	N/A	AF193278	Liitsola et al., [49]
NY5CG	Gln	**Leu**	Ser	0.018	AIDS	M38431	Willey et al., [50]
89.6	**Pro**	Val	Ser	0.049	AIDS	U39362	Collman et al., [51]
WEAU160	**Pro**	**Leu**	Ser	0.006	N/A	U21135	Unpublished
1299_d22	His	Val	**Pro**	0.018	N/A	AY308761	Bernardin et al., [52]
1006_08	**Pro**	Val	Ser	0.049	Acute infection	AY331284	Bernardin et al., [53]
1058_08	**Pro**	Val	Ser	0.049	Acute infection	AY331294	Bernardin et al., [53]
PRB959_03	Gln	Val	**Pro**	0.018	Acute infection	AY331296	Bernardin et al., [53]
RU128005	Gln	Val	**Pro**	0.018	N/A	AY682547	Unpublished
98USHVTN3605c9	**Pro**	Val	Ser	0.049	N/A	AY560108	Unpublished
PCM013	Glu	**Leu**	Ser	0.018	N/A	AY561237	Unpublished
50333-03	Gln	**Leu**	Ser	0.018	LTS	U30750	Iversen et al., [10]
931395-04	**Pro**	Val	Ser	0.049	AIDS	U30775	Iversen et al., [10]
LA-09	Gln	**Leu**	**Pro**	0.006	AIDS	U30785	Iversen et al., [10]

a; Sixteen of 164 Clade B HIV-1 *rev *sequences screened from the Los Alamos National Laboratory HIV Database and other published studies [10] contained Pro-74, Leu-104 and/or Pro-106 mutations. Underlined boldface indicates the presence of one or more of these rare amino acid changes.

b; Amino acid frequency was calculated by dividing the number of sequences with the amino acid, or the particular amino acid combination, by the total number of sequences analyzed (n = 164).

c; Patient MA, identified as a LTS with attenuated *rev *alleles in a previous study by Iversen et al., [10] was included for comparison.

d; LTNP, long-term nonprogressor; LTS, long-term survivor; AIDS, acquired immune deficiency syndrome; N/A, not available.

Rev is a highly structured protein [reviewed in [20,21]]. Biochemical and structural studies identified an α-helix at aa 8 to 26, and another at aa 34 to 59 spanning the NLS/RBD, separated by a Pro-rich region at aa 27 to 39, which folds into a helix-loop-helix structure where intramolecular contacts between the 2 α-helices are facilitated by hydrophobic interactions [reviewed in [20]]. The Rev RBD within the latter α-helix interacts specifically with an internal loop of the RRE through major groove interactions [33]. The C-terminal region of Rev is thought to be more flexible. However, a discontinuous epitope of a Rev-specific monoclonal antibody was mapped to aa 10 to 20 and 95 to 105 by protein foot printing, suggesting that the α-helices are in close proximity to the Rev C-terminus [34,35], and suggesting a role for the C-terminus in stabilizing native Rev structure. Thus, aa changes occurring at the Rev C-terminus or elsewhere such as Pro-74, Leu-104 and/or Pro-106 could potentially affect Rev structure and thus, Rev/RRE binding. Proline provides exceptional conformational rigidity to proteins. Thus, It is possible that Pro-74 and/or Pro-106 may impede RRE binding by altering native Rev structure.

### Rev derived from D36, but not C64, has impaired function

**Figure 3:**
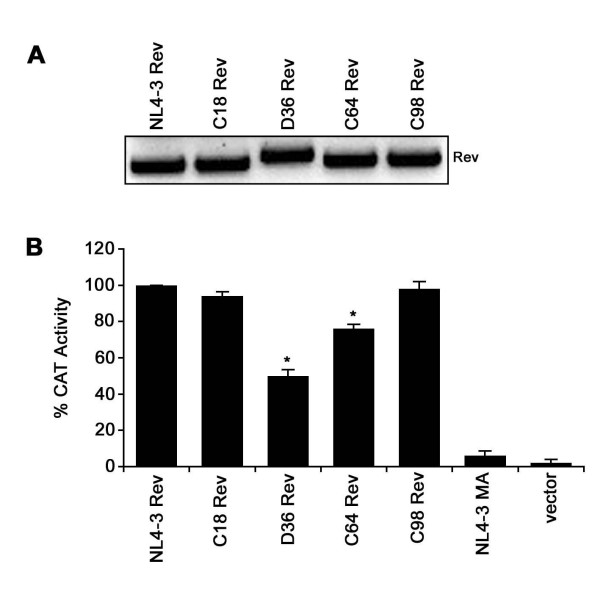
**Analysis of Rev protein expression and function in mammalian cells**. Rev function was examined by co-transfection of CEM cells with pcDNA3.1-Rev plasmid and the Rev-dependent pDM128 CAT expression plasmid [31], as described in Materials and Methods. Cells co-transfected with pDM128 and pcDNA3.1 expressing HIV-1_NL4-3 _Matrix protein or empty pcDNA3.1 vector were included as negative controls. Rev protein expression was determined by Western blotting with sheep anti-Rev polyclonal antisera (A). CAT activity in cell lysates was quantified and normalized to CAT activity in lysates of CEM cells co-transfected with pDM128 and NL4-3 Rev (B). Values shown are means of triplicate transfections. Error bars represent standard deviations. Results are representative of three independent experiments. *P < 0.01, Student's *t *test.

To determine whether SBBC Revs have impaired function, D36, C64, C18 and C98 Revs were subcloned into the pcDNA3.1 expression vector. Western blot analysis of Rev protein expression using sheep polyclonal anti-Rev antiserum showed equivalent levels of Rev in lysates of transfected CEM cells (Fig. 3A). Rev function in mammalian cells was investigated using the Rev-dependent reporter plasmid pDM128 [31], which expresses the chloramphenicol acetyltransferase (CAT) gene in the presence of Rev, as described previously [36] (Fig. 3B). In this assay, the Rev expression plasmids were first titrated to determine an amount to use that was within the linear response range of the assay (data not shown). Levels of CAT activity were compared to those present in lysates of cells co-transfected with pDM128 and HIV-1_NL4-3 _Rev. Cells cotransfected with pDM128 and empty pcDNA3.1 vector or pcDNA3.1 expressing HIV-1_NL4-3 _Matrix protein were included as negative controls. Levels of CAT activity in lysates of cells transfected with C18 or C98 Revs were not significantly different to those in lysates of cells transfected with HIV-1_NL4-3 _Rev. In contrast, levels of CAT activity in lysates of cells transfected with C64 or D36 Revs were reduced by approximately 20% and 50%, respectively (P < 0.01). Similar results were obtained using 293 cells (data not shown). In addition, similar results were obtained using a Rev-dependent HIV-1 *env *reporter system as a measure of Rev function, as described previously [37] (data not shown). These data suggest efficient Rev function by C18 and C98 Revs, a modest reduction in the activity of C64 Rev, but significant impairment in the activity of D36 Rev.

### Amino acid sequences associated with impaired D36 Rev function

Efficient C18 and C98 Rev function is consistent with results of the Rev/RRE binding studies that showed efficient RRE binding by these Revs (Fig. 2). However, the modest or significant impairment in C64 or D36 Rev activity, respectively, is discrepant with results of the Rev/RRE binding studies that showed equivalent reductions in RRE binding by these Rev variants (Fig. 2). Therefore, in C64 Rev, the reduced levels of RRE binding appear to be sufficient for the majority of Rev function to be retained. Additional sequence changes that differentiate C64 and D36 Revs are likely to impair D36 Rev function. Longitudinal sequence analysis showed that the presence of an unusual 13 aa extension at the D36 Rev C-terminus was the only genetic alteration that consistently differentiated D36 Rev from C64 Rev (Fig. 1), [see also Additional file 1]. The otherwise isogenicity of the D36 and C64 Revs used in the functional studies identifies the C-terminal 13 aa extension as the primary determinant underlying impaired D36 Rev function.

It is presently unclear how this sequence alteration may affect D36 Rev function, but the additional 13 aa at the Rev C-terminus may affect Rev structure. One hypothesis is that such structural changes may interfere with the recruitment of cellular proteins to the NES such as eIF-5A [38], nucleoporins including Rip/Rab [39-43] and CRM1/exportin 1 [44-46], which could potentially affect nuclear export. The presence of Pro at position 74 immediately N-terminal to the Rev NES may induce further structural changes contributing to this interference, which might also account for the modest reduction in C64 Rev activity. Further studies are required to fully elucidate the importance of amino acid alterations that impair D36 Rev function.

## Conclusion

In this study, we demonstrate reduced capacity of persistent and dominant Rev variants isolated from a subset of SBBC members to bind the RRE, which was associated with unique *rev *alleles carrying rare amino acid substitutions at 3 highly conserved positions outside the RBD; Gln to Pro at position 74 immediately N-terminal to the Rev activation domain, and Val to Leu and Ser to Pro at positions 104 and 106 at the Rev C-terminus, respectively. However, decreases in Rev/RRE binding *per se *were not sufficient to attenuate Rev function. This conclusion is supported by studies of C64 Rev, which had significantly reduced RRE binding but only modestly reduced Rev function. Additional sequence changes present in D36 Rev attenuated Rev function. This was mapped to an unusual 13 aa extension at the Rev C-terminus, which was the only genetic change that distinguished C64 and D36 *rev *alleles. This genetic alteration may alter structural properties of Rev that are required for optimal Rev function. Together, our data suggest that Rev function, not Rev/RRE binding may be rate limiting for HIV-1 replication.

It is presently unclear whether attenuated D36 Rev function *in vitro *equates to attenuated Rev function *in vivo*, and indeed whether attenuated Rev function contributed to slow progression of HIV-1 infection in this subject. Extrapolation of these *in vitro *findings to an *in vivo *role for attenuated D36 *rev *alleles is difficult, since this subject and other SBBC members are infected with virus containing gross *nef*/LTR deletions which have been shown to contribute significantly to viral attenuation in this cohort [1,4,27]. Furthermore, the attenuated properties of D36 and C64 Revs did not distinguish SBBC LTNP from SP. In fact, among the SBBC subjects studied here, D36 had the most attenuated *rev *alleles yet the most progressive HIV-1 infection, suggesting that any effect that attenuated *rev *alleles may have *in vivo *is likely to be dependent on other viral and/or host factors. Nonetheless, our results support those of a previous study that showed attenuated Rev function in an asymptomatic individual [10], and those of another study that showed reduced Rev function among *rev *alleles with naturally occurring sequence variations [26], raising the possibility that attenuated Rev function may contribute, at least in part, to viral attenuation and slow HIV-1 progression in D36. However, in contrast to these studies where attenuated Rev function was mapped to mutations in the activation domain [10,26], attenuated Rev function in D36 was mapped to the Rev C-terminus.

In sum, these findings provide new genetic and mechanistic insights important for Rev function. In addition, attenuated *rev *alleles may contribute to viral attenuation and long-term survival of HIV-1 infection in a subset of SBBC members. A better understanding of viral determinants other than *nef*/LTR that contribute to HIV-1 pathogenicity (or lack thereof) in SBBC members may provide additional mechanistic insights important for controlling HIV-1 infection *in vivo*.

## Methods

### Rev cloning and sequencing

Full-length HIV-1 Rev clones containing the first and second Rev coding exons were generated from genomic DNA of patient PBMC samples by PCR using Expand high fidelity DNA polymerase (Roche Diagnostics, Basel, Switzerland) as follows; The first Rev coding exon was amplified using primers 5RevE2 (5'-GGGTGTCGACATAGCAGAATAG-3'; corresponding to nt positions 5781 to 5802 of HIV-1_NL4-3_) and 3RevE2 (5'-CTGCTTTGATAGAGAAGCTTG-3'; corresponding to nt positions 6024 to 6044 of HIV-1_NL4-3_) that spans a *Sal*I restriction site 5' to the Rev start codon. The second Rev coding exon was amplified using primers 5RevE3 (5'-CCACCTCCCAATCCCGAGGGG-3'; corresponding to nt positions 8371 to 8391 of HIV-1_NL4-3_) and 3RevE3 (5'-CTAGGTCTCGAGATACTGCTC-3'; corresponding to nt positions 8879 to 8898 of HIV-1_NL4-3_) that spans an *Xho*I restriction site 3' to the Rev stop codon. To avoid sequence resampling, six independent PCRs of Rev exon 1 or Rev exon 2 coding sequence were pooled prior to 3-way ligation in pGEM (Promega, Madison, WI) using *Sal*I and *Xho*I restriction sites and blunt end ligation to link the Rev exon 1 and Rev exon 2 coding sequences.

Ten independent Revs cloned from each PBMC sample were sequenced using a SequiTherm EXCEL II DNA sequencing kit (Epicenter Technologies, Madison, WI) and a model 4000L LI-COR DNA sequencer (LI-COR, Lincoln, NE). Predicted aa sequences were deduced from nucleotide sequences, and aligned and analyzed using DNAMAN software (Lynnon, Quebec, Canada).

### Rev/RRE binding assays

His-tagged Rev proteins derived from SBBC *rev *alleles were produced, purified and quantified using the pET bacterial expression system (Novagen, Madison, WI), according to the manufacturers' protocol. The ability of His-tagged Rev proteins to bind the RRE was quantified by electrophoretic mobility shift assays with [^32^P]-labelled RNA transcripts bearing the RRE, as described previously [47]. Briefly, binding reactions consisted of excess [^32^P]-labelled RNA and increasing concentrations of His-tagged Rev protein (0, 0.05, 0.25, 0.40 or 0.50 μM) in 10 μl binding buffer [10 mM HEPES/KOH (pH 7.6), 150 mM KCl, 2 mM MgCl_2_, 0.5 mM EGTA, 1 mM dithiothreitol, 20% (vol/vol) glycerol, 3.2 μg *E. coli *tRNA]. Reactions were incubated on ice for 10 min, then applied to 5% (wt/vol) nondenaturing polyacrylamide gels containing 100 mM Tris borate (pH 8.3), 1 mM EDTA, and 3% (vol/vol) glycerol and run at 4°C followed by autoradiography and phosphorimager analysis.

### Rev function assays

To facilitate Rev protein expression in mammalian cells, SBBC *rev *alleles were subcloned into the pcDNA3.1 expression vector (Invitrogen, Carlsbad, CA). Rev function in mammalian cells was quantified using the Rev-dependent reporter plasmid pDM128, which expresses the CAT gene from an intron bearing the RRE in the presence of HIV-1 Rev [31]. Briefly, CEM cells were cotransfected with 4.0 μg pDM128, 0.75 μg pcDNA.1-Rev plasmid and 0.25 μg pEGFP plasmid to control for transfection efficiency. The Rev expression plasmids were titrated first to determine an amount to use that was within the linear response range of the assay (data not shown). After minor volume adjustments for small variations in transfection efficiency, cell lysates were prepared at 72 h post-transfection and assayed for CAT activity as described previously [36].

### Western blot analysis

Lysates were prepared from CEM cells that were transfected as described above, separated in 12% (wt/vol) SDS-polyacrylamide gels, and transferred to nitrocellulose membranes, as described previously [37]. Blots were probed with a 1:500 dilution of sheep anti-Rev polyclonal antisera (ICN). Rev proteins were visualized using horseradish peroxidase-conjugated anti-sheep immunoglobulin G antibody and enhanced chemiluminescence (Promega).

### Nucleotide accession numbers

The *rev *nucleotide sequences reported here have been assigned GenBank accession numbers EF634153 to EF634156.

## Competing interests

The author(s) declare that they have no competing interests.

## Authors' contributions

MJC and PRG designed the study, MJC and LC performed the experiments, MJC and PRG analyzed the data and wrote the paper, SLW contributed to the experimental design and data analysis.

## Supplementary Material

Additional file 1 Consensus Rev amino acid sequences from sequential SBBC blood samples. Each sequence represents the consensus of 10 independent Rev clones from each time point. Amino acid alignments are compared to Rev from HIV-1_NL4-3_. Dots indicate residues identical to HIV-1_NL4-3 _Rev, and dashes indicate gaps. Note the persistence of a dominant *rev *allele in each subject over the time course studied. Click here for file
