# Supplementary figures and images for: Persistence of attenuated HIV-1 rev alleles in an epidemiologically linked cohort of long-term survivors infected with nef-deleted virus
Source: Retrovirology. 2007 Jul 1;4:43. doi: 10.1186/1742-4690-4-43 (PMC1933581; doi:10.1186/1742-4690-4-43)

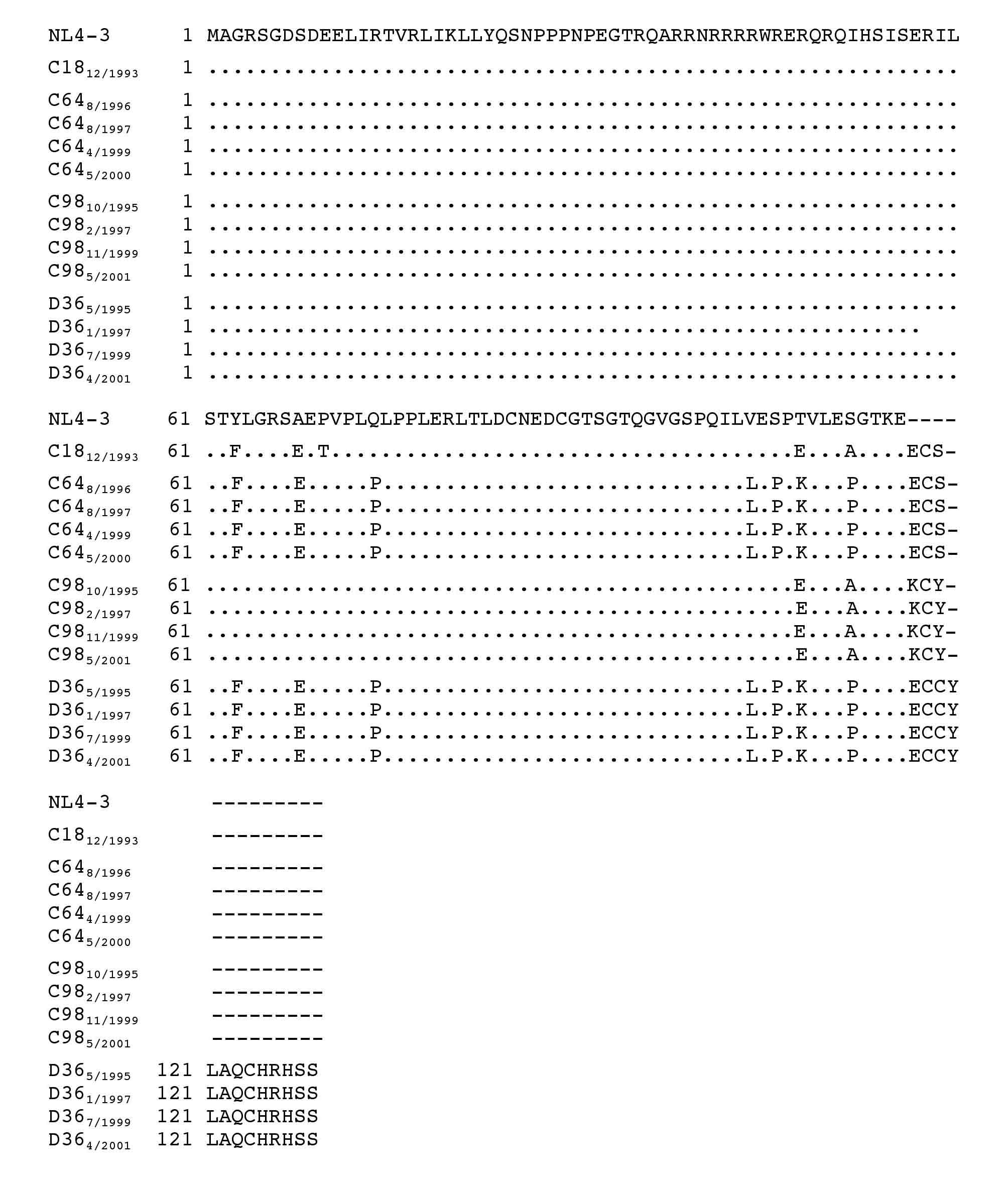

Supplement: Additional file 1 — Consensus Rev amino acid sequences from sequential SBBC blood samples. Each sequence represents the consensus of 10 independent Rev clones from each time point. Amino acid alignments are compared to Rev from HIV-1NL4-3. Dots indicate residues identical to HIV-1NL4-3 Rev, and dashes indicate gaps. Note the persistence of a dominant rev allele in each subject over the time course studied. [file 1742-4690-4-43-S1.jpeg]
